# A Compact Band-Pass Filter with High Selectivity and Second Harmonic Suppression

**DOI:** 10.3390/ma6125613

**Published:** 2013-12-03

**Authors:** Ramona Cosmina Hadarig, Maria Elena de Cos Gomez, Fernando Las-Heras

**Affiliations:** Signal Theory and Communications Area, Department of Electrical Engineering, University of Oviedo, Multi-Purpose Building, Module 8, Gijón 33203 (Asturias), Spain; E-Mails: medecos@tsc.uniovi.es (M.E.C.G.); flasheras@tsc.uniovi.es (F.L.-H.)

**Keywords:** band-pass filter, artificial magnetic conductor, dispersion diagram, second harmonic suppression, transmission coefficient

## Abstract

The design of a novel band-pass filter with narrow-band features based on an electromagnetic resonator at 6.4 GHz is presented. A prototype is manufactured and characterized in terms of transmission and reflection coefficient. The selective passband and suppression of the second harmonic make the filter suitable to be used in a C band frequency range for radar systems and satellite/terrestrial applications. To avoid substantial interference for this kind of applications, passive components with narrow band features and small dimensions are required. Between 3.6 GHz and 4.2 GHz the band-pass filter with harmonic suppression should have an attenuation of at least 35 dB, whereas for a passband, less than 10% is sufficient.

## 1. Introduction

In recent years, emerging applications have continued to challenge radio frequency (RF)/microwave filters’ designers with stringent simultaneous requirements such as high performance, light weight, low cost and miniaturization. As the electromagnetic spectrum is limited and has to be shared, small sized band-pass filters with narrow frequency response and high selectivity are used to confine the signals within assigned spectral limits and to reject the noise and interferences from adjacent channels.

To minimize the filter size, a practical strategy is to reduce the resonator circuit by modifying its physical structures [1,2,3,4,5,6,7]. Starting from the conventional parallel coupled band-pass filter [8,9,10,11] which has a simple synthesis procedure, and ending with U-shape resonators and open loops [12,13], hairpin filters [14] has helped progress in size reduction. However, with the rapid evolution of modern communication systems [15], the sizes of these resonators are still not small enough to be used.

In this work, a novel low thickness band-pass filter based on a simple and compact size electromagnetic resonator without vias is presented. The novelty of this contribution relies on taking advantage of the predicted electromagnetic band-gap (EBG) properties of a resonator unit-cell with improved performance (by means of its dispersion diagram) to achieve the intended band-pass filter behavior. Moreover, the filter exhibits a narrow passband, high out-of-band rejection level, and second harmonic suppression. The paper is organized as follows. Section 2 outlines the study of the unit-cell resonator in terms of dispersion diagram. In Section 3, the design of the filter is presented based on the unit-cell resonator. Also, the possibility of suppressing of the second harmonic is discussed. Two filters are manufactured and measurements concerning the transmission coefficient are provided to show their performance results.

## 2. Resonant Element Design

The first step in the design of the band-pass filter (henceforth referred to as BPF) is the design of the resonant element. In [16], the resonant element—replicated to model an infinite resonant structure—is characterized as an Artificial Magnetic Conductor (AMC) material [17,18,19,20] and the in-phase reflection property was studied. This feature enables efficient radiation for antennas placed closed to the periodic structure. In this contribution the resonant structure is seen from another point of view: as a material that has the possibility to block the propagation of electromagnetic waves in certain frequency bands and guide them in a desired direction [*i.e.*, electromagnetic band-gap (EBG)].

**Figure 1:**
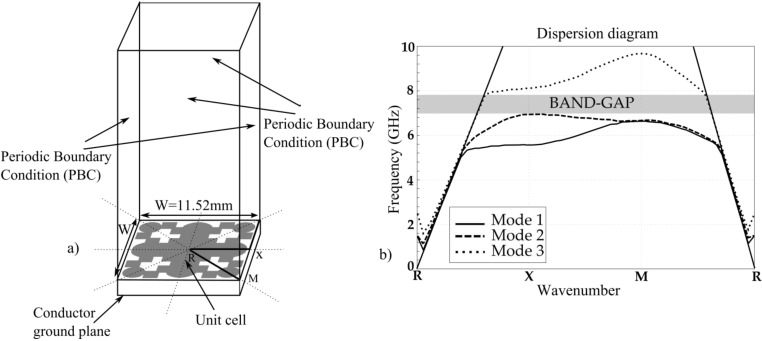
(**a**) Unit-cell resonator. The applied boundary conditions with the irreducible Brillouin triangle; and (**b**) Dispersion diagram of the periodic structure.

EBG structures [21,22] exhibit allowed and forbidden bands of modes’ propagation and can be characterized by the dispersion diagram. The dispersion diagram for a unit-cell of period W will show the relation between the wave number and frequency, giving information about the propagating modes and band-gaps that can potentially exist between such modes [23,24]. The eigenmode solver of High Frequency Structural Simulator (HFSS) together with proper boundary conditions along the sides of the unit-cell (resembling an infinite structure) is used in order to determine the dispersion diagram of the structure (see Figure 1a). Due to symmetry of the unit-cell, the dispersion diagram is computed along the lines connecting the R, X and M points, to form the irreducible Brillouin triangle. Thus the dispersion analysis of periodic structures is reduced to find only the propagation modes in the direction of the vectors of the irreducible Brillouin triangle. The dispersion characteristics, *i.e.*, the position and width of the band-gap and the frequency of the propagating modes are primarily defined by the geometry of the unit-cell. ARLON25N with relative dielectric permittivity 3.28, loss tangent less than 0.0025 and a thickness of 0.762 (30 mils) is used as dielectric substrate. The unit-cell dimensions are *W* × *W* = 11.52 mm × 11.52 mm and its geometry exhibits four symmetry planes. The metallization thickness is 18 μm.

The dispersion diagram for three lowest modes is depicted in Figure 1b. The first mode propagates in the frequency range from 5 to 6.6 GHz, whereas the second mode propagates from 5 to 7 GHz. The resonance frequency takes place between the first and second mode, being around 6 GHz. Moreover, the bandgap is presented in a frequency range from 7 to 7.8 GHz. Within the bandgap, the electromagnetic waves cannot propagate at any direction in the EBG structure.

## 3. Filter Design

With the purpose of designing a band-pass filter having the same size as the unit-cell resonator, the propagation mode paths along the R-X-M triangle and predicted by the dispersion diagram could be followed. The frequency of the modes propagating along the edges of the Brillouin triangle have to be taken into account to define the filter passband, whereas the band-gap can be used to achieve the filter stopband. The computed dispersion diagram shows the propagation of the first mode in the frequency range from 5 to 6.6 GHz. In the present work, only the (M-R) region and part of the (X-M) region from the dispersion diagram were considered due to the fact that the authors’ goal was to obtain a selective band-pass filter.

**Figure 2:**
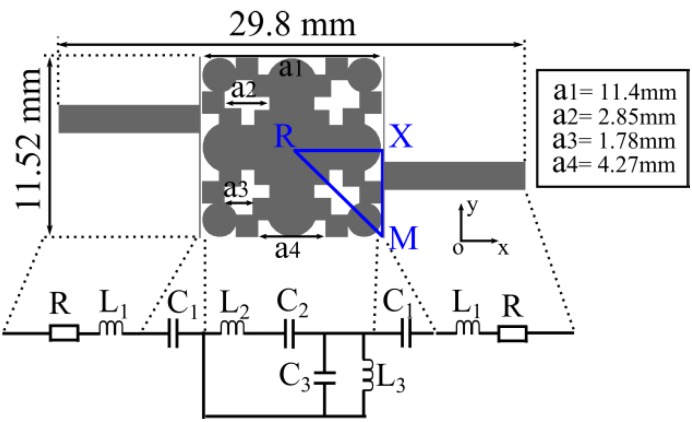
Layout of the band-pass filter (BPF) with its characteristic circuit model.

The distance between the metallization edge and the unit-cell edge influences the band-gap position. More precisely when the mentioned distance increases, the band-gap shifts to a higher frequency band whereas its width increases. The variation of the *a*2 and *a*3 parameters (see Figure 2) has the following effect: as *a*2 and *a*3 increase the band-gap shifts to a lower frequency band whereas the width of the band-gap increases. When *a*1 and *a*4 parameters decrease, meaning that the whole unit-cell size decreases, the band-gap shifts to a higher frequency band and its width increases. The thickness of the dielectric substrate also has an influence on the band-gap position. Increasing the thickness, the band-gap shifts to a lower frequency band and in the same time its width increases.

The filter consists of a unit-cell resonator and two narrow lines coupled to the resonator. Each narrow line has a width of 0.1 mm and a length of 11.52 mm. The two narrow lines are placed symmetrically with respect to the unit-cell resonator. The gap between the coupling narrow line and unit-cell resonator is 0.1 mm. The filter is excited by a pair of non-orthogonal input/output 50 Ω microstrip feeding lines of 1.8 mm width and is printed on an ARLON25N substrate with a thickness of 30 mils, relative dielectric permittivity of 3.28, cooper thickness 18 μm and loss tangent 0.0025. The layout of the filter in 6.4 GHz frequency band is presented in Figure 2.

In order to determine the characteristics of the BPF, two approaches could be followed. The first one is to model the BPF using its equivalent circuit (or at least a simplified version) as can be seen in Figure 2. The resonator unit-cell can be model based on transmission line theory [25] providing that the unit-cell size is electrically small enough. The presented unit-cell is λ_0_/5 so it can be considered not so small and being at the limit of application of such a model. In any case the model would comprise a series connection (L_2_ and C_2_) in parallel with the equivalent impedance of the parallel L_3_ and C_3_ components. In series with the mentioned circuit the capacitance C_1_ (formed between the resonator and each narrow coupling line) and L_l_ and R components (modeling the transmission line) are placed. The second approach consists in explaining the physical phenomena under the filter behavior. To achieve this aim the modes propagation in the structure should be explained based on dispersion diagram so the second approach is followed.

**Figure 3:**
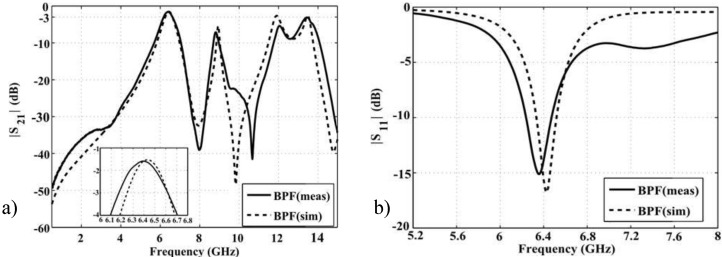
(**a**) Simulation *vs*. measurement: Transmission coefficient of the BPF; (**b**) Simulation *vs*. measurement: Reflection coefficient of the BPF.

The dimensions of the filter are 29.8 × 11.52 × 0.762 mm^3^ (0.64λ_0_ × 0.25λ_0_ × 0.016λ_0_, λ_0_ is the free space wavelength, λ_0_ = 47 mm at 6.4 GHz) considering the input/output feeding lines and 11.92 × 11.52 × 0.762 mm^3^ (0.25λ_0_ × 0.25λ_0_ × 0.016λ_0_) without the input/output feeding lines. From simulation results in Figure 3a, the 3dB passband of BPF without harmonic suppression goes from 6.25 to 6.62 GHz, meaning 5.75% fractional bandwidth at the center frequency 6.44 GHz. The minimum insertion loss is 1.7 dB whereas the maximum return loss value is greater than 17 dB (see Figure 3b).

Taking into account the position of the feeding lines with respect to the unit-cell geometry, the propagation follows a quasi-diagonal path, which coincides with the (M-R) region from the dispersion diagram as being together with part of the (X-M) region. Furthermore, from the (M-R) region, as well as part of the (X-M) region of the dispersion diagram resulted in the first mode propagating in a frequency spectrum of between 6 and 6.6 GHz. This band corresponds to the electromagnetic wave propagation through the BPF geometry whereas from 6.94 to 7.9 GHz the structure does not allow any mode propagation which corresponds to the stopband frequency region of the BPF.

In Table 1, the 3 dB bandwidth together with the quality factor are presented. In a band-pass filter, the overall width of the passband between the upper and lower 3 dB levels of the filter determines the quality factor Q. The lower the value of the Q factor, the wider the bandwidth and, consequently, the higher the Q factor, the narrower and more selective the filter.

**Table 1:** Simulated band-pass filter quality factor.

Prototype	3 dB		*f*_c_	BW(3 dB)		Q	Size (mm × mm)	Size (mm × mm)
	*f*_low_(GHz)	*f*_high_(GHz)		GHz	%			
BPF	6.25	6.62	6.44	0.37	5.57	17.40	29.80 × 11.52 *	11.92 × 11.52
3-cells-oy BPF	6.45	6.61	6.52	0.16	2.45	40.75	29.80 × 34.56 *	11.92 × 34.56
3-cells-ox BPF	6.55	6.67	6.62	0.12	1.81	55.16	52.84 ×11.52*	34.96 × 11.52
Square BPF	6.13	7.30	6.70	1.17	17.4	5.72	29.80 ×11.52*	11.92 × 11.52

* Considering the input/output feeding lines.

**Figure 4:**
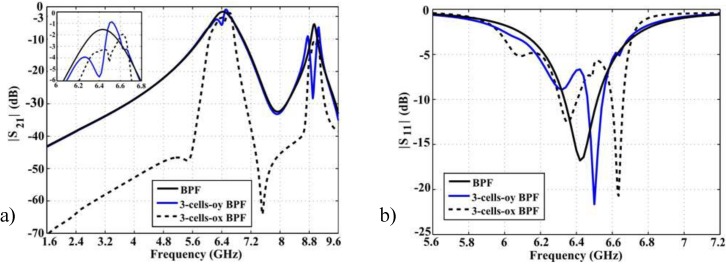
(**a**) S21 simulation results; and (**b**) S11 simulation results.

According to the analysis shown in Table 1, if three resonators are cascaded in the OY direction (henceforth referred to as 3-cells-oy BPF) the 3 dB passband goes from 6.45 to 6.61 GHz, meaning 2.45% fractional bandwidth at the center frequency 6.52 GHz, whereas in the OX direction (filter henceforth referred to as 3-cells-ox BPF) the 3 dB passband goes from 6.55 to 6.67 GHz, meaning 1.8% fractional bandwidth at the center frequency 6.62 GHz. Moreover, using three cells in the direction of the current flow, the filter becomes more selective (see Figure 4a). The increment in the number of unit-cells in the OY direction has an influence only in the passband of the filter, which becomes narrower; meanwhile the slope in the stop bands remains the same. The benefit of the novel BPF over the square shaped resonator BPF is a quality factor three times greater. Figure 4b shows that the return loss increases with the number of unit-cells.

In order to suppress the second harmonic, several techniques have been reported in literature. In [26] two quarter wavelength open ended stubs are attached at the edges of the resonators in the main coupling path in order to have harmonic suppression, but this implies additional circuit elements against compact filter design. Another interesting alternative, such as using a continuous modulation in the coupled section of the filter [27,28] and using Koch fractal geometry [29], have also been considered, but both are time consuming due to the need of many parameters optimization. In this contribution, defected ground structures (DGS) are used to act as a low-pass filter. The slots are placed in the ground plane, directly under the input/output feeding lines [30].

**Figure 5:**
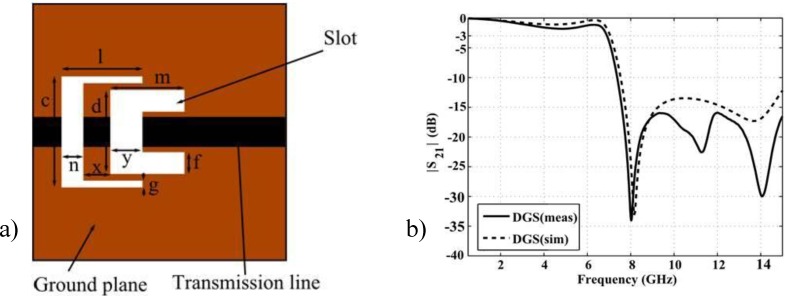
(**a**) Topology of defected ground structures (DGS) section (bottom view); and (**b**) transmission coefficient of DGS section.

The dimensions of the slots were chosen as *c* = 6.6 mm, *d* = 5 mm, *f* = 1.3 mm, *g* = 0.4 mm, *l* = 4.8 mm, *m* = 4.4 mm, *n* = 1.3 mm, *x* = 1.6 mm, *y* = 1.9 mm (see Figure 5a). The conductor strip of the microstrip line on the top plane has a width of 1.8 mm, corresponding to 50 Ω characteristic impedance.

From simulation results in Figure 5b, the DGS structure under the microstrip transmission line exhibits a 3 dB cutoff frequency at 7.15 GHz and a center frequency of the stop band at 8.25 GHz with a maximum attenuation of 33 dB.

**Figure 6:**
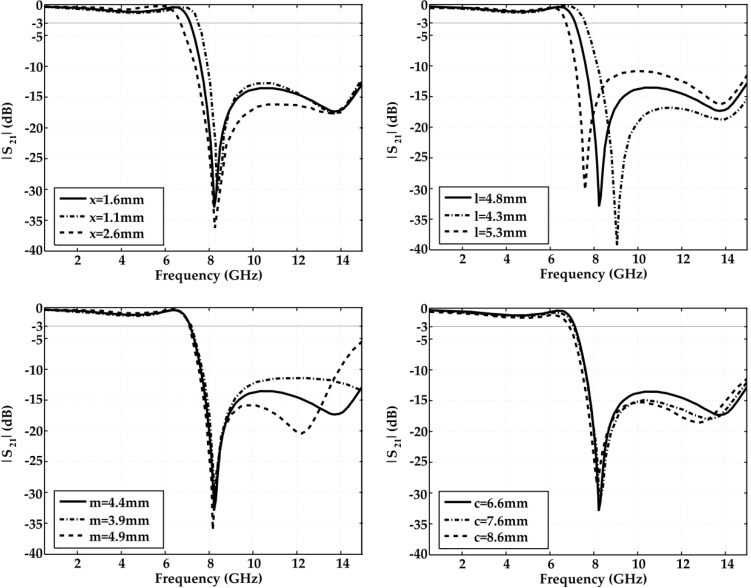
Parametric analysis of the DGS.

The dimensions of the slots placed in the ground plane influence the 3 dB cutoff frequency and the attenuation. Figure 6 shows the parametric study of the most important parameters “*x*”, “*l*”, “*m*” and “*c*”. Only one parameter is changed at a time during the analysis. By increasing the “*x*” parameter, the 3 dB cutoff frequency decreases whereas the attenuation is almost the same at 8.25 GHz. The “*l*” parameter controls both the attenuation at the center frequency of the stopband and the 3 dB cutoff frequency. On decreasing “*l*”, the center frequency of the stopband, the attenuation and the 3 dB cutoff frequency increase. The parameters “*c*” and “*m*” influence only the 3 dB cutoff frequency. The 3 dB cutoff frequency decreases when the parameters “*c*” and “*m*” decrease.

## 4. Results

**Figure 7:**
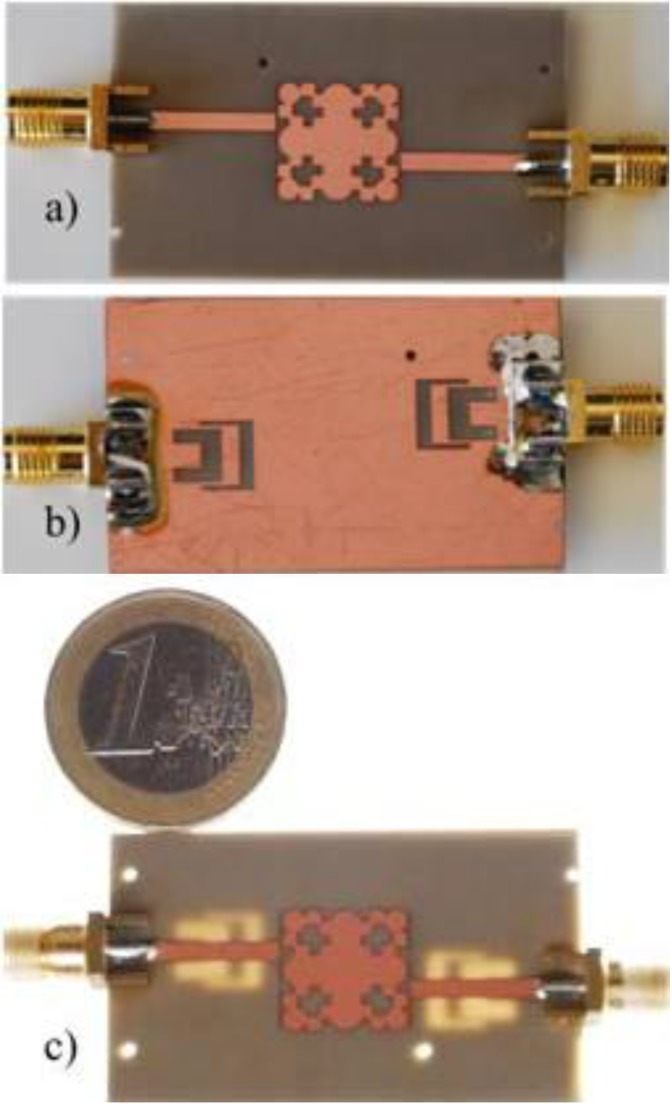
Manufactured prototype band-pass filter with second harmonic suppression (BPF-DGS) (**a**) top view; (**b**) bottom view; and (**c**) cross-section view.

Prototypes of the band-pass filter with second harmonic suppression (henceforth referred to as BPF-DGS) and without harmonic suppression using one unit-cell resonator, have been manufactured using laser micromachining (see Figure 7).

**Figure 8:**
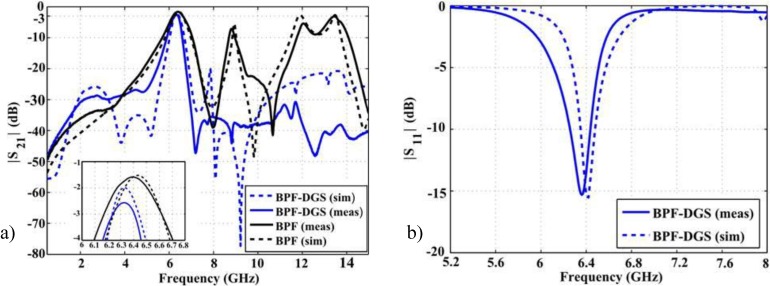
(**a**) S21 simulation and measurement results; and (**b**) S11 simulation and measurement results.

The results of measured transmission coefficient for the BPF-DGS and BPF prototypes are depicted in Figure 8a. The measured 3 dB bandwidth goes from 6.16 to 6.62 GHz, signifying 460 MHz (7.18%) for the BPF prototype. There is good agreement between measurement and simulation results for the filter without harmonic suppression.

Regarding the BPF-DGS prototype, a 3 dB passband of 210MHz (3.32%) at the center frequency 6.32 GHz is obtained in simulation whereas, in measurements, 175 MHz (2.76%) passband at 6.33 GHz resulted. In the stopband regions some slightly differences between measurement and simulation can be observed. The fact that commercial MoM software considers infinite extension of the dielectric substrate, together with manufacturing process tolerances and cables’ and connectors’ losses, explains the differences in the stopband region. Nevertheless, the simulations and measurement results are in good agreement and they meet the application requirements in the C band range. The filter with DGS exhibits approximately 50% less pass bandwidth than the BPF without harmonic suppression because of the reduced coupling generated.

The absence of multilayer substrates and vias make the filters suitable to be used not only in satellite/terrestrial communication applications, but also in wearable applications where the prototypes could be shape adapted and bended. From Table 2, it can be easily seen that the size of the proposed BPF is minimized compared to band-pass filters with similar performance and substrate as used in [31,32,33,34], whereas the BPF-DGS shows a higher quality factor.

**Table 2:** Comparison with other band-pass filters.

Prototype	3 dB		*f*_c_	BW(3 dB)		Q	Size (mm × mm)	Size λ—wavelength at the corresp. *f*_c_
	*f*_low_(GHz)	*f*_high_(GHz)		GHz	%			
BPF	6.25	6.62	6.44	0.37	5.75	17.40	29.8 × 11.52 *	0.63λ × 0.25λ *
							11.92 × 11.52	0.25λ × 0.25λ
BPF-DGS	6.23	6.40	6.33	0.17	2.76	36.38	29.8 × 11.52	0.63λ × 0.25λ
[31]	5.46	5.84	5.65	0.38	6.72	14.80	46.2 × 11.5	0.87λ × 0.21λ
[35]	5.58	5.83	5.71	0.25	4.46	22.80	40 × 10	0.76λ × 0.19λ
[35]	5.39	6.02	5.71	0.63	11.14	9.06	40 × 10	0.76λ × 0.19λ
[36]	5.88	6.12	6	0.24	4.05	25	15 × 13	0.3λ × 0.26λ
[32]	6.63	6.96	6.8	0.33	4.9	20.6	20 × 22	0.45λ × 0.5λ
[33]	5.09	5.35	5.25	0.26	4.9	20.2	45 × 14	0.79λ × 0.25λ
[34]	5.14	5.38	5.25	0.24	4.57	21.8	19.8 × 17.9	0.35λ× 0.31λ
[37]	4.78	5.61	5.2	0.83	16	6.26	26.3 × 9.9	0.45λ × 0.17λ
[38]	5.54	5.86	5.7	0.32	5.6	17.81	26 × 8	0.5λ × 0.15λ
[39]	1.36	1.49	1.42	0.13	9	10.9	22.14 × 5.08	0.1λ × 0.023λ
[40]	2.84	4.2	3.4	1.36	40	2.5	20 × 15	0.22λ × 0.17λ

* Considering the input/output feeding lines.

The miniaturized BPF-DGS is demonstrated at 6.33 GHz, which is a higher frequency than the reported filter in [35]. If the proposed filter would be made to operate in the same frequency band as in [35], the filter would have a smaller size compared to [35].

In [37], the proposed filter renders a quality factor of 6.26, which is approximately three or six times lower compared to 17.4 of the BPF and 36.38 of the BPF-DGS. In [39], the upper conducting layer of the filter is connected through the bottom ground plane using vias. Even though in [39] a 0.1λ_0_ × 0.023λ_0_ miniaturized band-pass filter is shown, with a lower quality factor, it has the disadvantage of high cost and a difficult manufacture process due to the use of vias which are vulnerable to environmental influences such as being insufficiently plated through or filled with solder. This may cause the delamination or cracking of the vias.

## 5. Conclusions

The design of a frequency selective band-pass filter with small dimensions and second harmonic suppression using an electromagnetic resonator has been presented. A prototype has been manufactured at 6.4GHz and characterized based on transmission loss measurements. Second harmonic suppression was obtained using a DGS topology. The filter presents a high selectivity with a sharp passband to stop band transition. The compact size, low cost, simple fabrication and integration with other components in the system make it appropriate for satellite/terrestrial communication and wearable applications.
